# Lightweight Security Transmission in Wireless Sensor Networks through Information Hiding and Data Flipping

**DOI:** 10.3390/s22030823

**Published:** 2022-01-21

**Authors:** Lan Zhou, Ming Kang, Wen Chen

**Affiliations:** School of Cyber Science and Engineering, Sichuan University, Chengdu 610065, China; zhoulan@stu.scu.edu.cn (L.Z.); mkang@stu.scu.edu.cn (M.K.)

**Keywords:** wireless sensor network, information hiding, pseudo-random function, distributed detection, likelihood ratio test

## Abstract

Eavesdroppers can easily intercept the data transmitted in a wireless sensor network (WSN) because of the network’s open properties and constrained resources. Therefore, it is important to ensure data confidentiality in WSN with highly efficient security mechanisms. We proposed a lightweight security transmission method based on information hiding and random data flipping to ensure that the ally fusion center (AFC) can achieve confidential data transmission over insecure open links. First, the sensors’ local measurements are coded into a customized binary string, and then before data transmission, some parts of the string are flipped by the sensors according to the outputs of a pre-deployed pseudo-random function. The AFC can recover the flipped binaries using the same function and extract the measurement hidden in the string, while the enemy fusion center (EFC) cannot distinguish flipped and non-flipped data at all, and they cannot restore the measurement correctly as long as one bit in the string is not correctly recovered. We proved the security and anti-interference of the scheme through both simulations and physical experiments. Furthermore, the proposed method is more efficient such that it consumes less power than traditional digital encryptions through real power consumption tests.

## 1. Introduction

As we all know, wireless sensor networks (WSNs) are currently widely employed in various fields, especially in military reconnaissance [1], drone monitoring [2], industrial control [3], transportation [4], etc., to collect various physical or environmental data. Sometimes, WSN is deployed in places that humans cannot reach or in enemy areas. Distributed detection is a common decision-making method in WSN, which means that multiple sensors are deployed in a target area to measure the physical state of a target in a decentralized manner. Each sensor makes a local decision based on its observations, and then it sends the decision to the Ally Fusion Center (AFC) through a wireless channel. The AFC utilizes all the received sensor data to make the final decision with regard to the target. However, as the sensor data are transmitted in a broadcast method, the Enemy Fusion Center (EFC) in the network can easily eavesdrop on the data, which is a serious threat to the confidentiality of the data transmissions in WSN. In addition, WSN is usually battery-powered to maintain its work. Therefore, the traditional encryption methods, which rely on complex calculations, are unsuitable for WSN. To extend the duration of sensor nodes and ensure the confidentiality of the data transmission, it is important to find a lightweight and safe data transmission scheme for WSN.

Faced with the above challenges, several lightweight encryption schemes have been proposed by researchers. Khashan et al. [5] proposed an automated and lightweight encryption scheme using a dynamic clustering technique which supports mobility in WSN, and it dynamically controls the complexity of the encryption process based on the currently limited resources. Cao et al. [6] proposed an improved identity-based encryption algorithm that lies between the traditional public-key encryption and identity-based public tweezers’ encryption, effectively simplifying the key generation process. In [7], the scheme first forwards all traffic packets in a disordered manner, using different network paths and protocols, and then distributes the traffic packets from one stream to another based on different encryption schemes. Usually, these schemes that rely on traditional encryptions to secure data confidentiality still face the computational burden of encryption and decryption.

On the other hand, several novel lightweight efficient schemes have been proposed. A physical layer-based security framework was proposed by Bashar et al. [8]. Assuming that the transmission channel of WSN is exposed to generalized K-fading, the framework uses cumulative distribution, optimal sensor, and round-robin scheduling schemes to reduce the probability of being eavesdropped. In [9], the authors designed a secure transmission scheme based on artificial noise. The transmitter designs the noise precoding matrix based on the known channel state information of the legitimate channel. The receiver can calculate the transmitted data from the probability distribution of channel state information, but the eavesdropper cannot. In [10], Jeon et al. proposed to explore the randomness of the wireless channel gains to encrypt the sensors’ local measurements with random flipping of the measured data. However, the scheme is only suitable for ideal situations, such that the instantaneous channel gains of sensors to the AFC all conform to the expected Rayleigh distribution, which is difficult to achieve in the real time-varying physical channels. In [11], Zhang et al. improved Jeon’s work. They optimized the flipping thresholds and let some sensors stay inactive to save transmission energy according to the sensors’ local detection confidence levels. In [12], Chen et al. designed an immune-based differential evolution algorithm to find the optimal rollover rate to minimize the fusion error of AFC while maintaining the fusion error of EFC at a high level. In [13], Yaacoub et al. proposed a clustering algorithm for grouping sensor nodes into cooperative clusters, and the sensor sends data to the parent node with minimal energy consumption and randomly flips bits based on channel state information to reduce the energy consumption and transmission time of the sensor data. In [14], the security transmission scheme is extended to a scenario with multiple quantization scales and candidate states. Currently, most of the schemes are designed based on the instantaneous channel state conformed to an expected ideal distribution. If the statistic of the channel state changes from time to time, it is very difficult to meet the required conditions, which seriously restricts the applications of these security models.

In this paper, we propose a secure data transmission scheme based on information hiding and simple data flipping. The basic idea is to first hide the local measurements into a customized binary string based on the principle of encoding hiding [15]. Then, the sensors and the AFC generate a random number sequence synchronously based on the output of a pre-deployed pseudo-random function, which determines the data flipping. Finally, the flipped binary strings along with error correction codes are sent to the AFC, which can recover the flipped strings and then extract the sensor observations from the strings. Our scheme has a simpler calculation process than traditional encryption and decryption algorithms, and it is more suitable for resource-constrained WSN.

The contributions of this paper can be summarized as follows:

(1) The proposed scheme has the anti-interference ability, and it can resist the change of channel environment and malicious attacks of EFC.

(2) The scheme is based on information hiding, which increases the data confidentiality, such that as long as one bit of the binary string is not correctly recovered, the EFC fails to withdraw the true measurements coded in the string.

(3) The energy consumption is lower than that of the traditional encryption method. Traditional encryption algorithms are relayed on complex key distribution, S-box confusion, and multiple rounds of iterative calculations. On the contrary, our scheme only needs simple data flipping, which is much more efficient.

(4) Groups of comparisons are carried to test the confidentiality and efficiency of our method in both simulations and real environments.

The rest of this article is organized as follows. Section 2 gives the system model. Section 3 gives a safety analysis. The computational complexity is discussed in Section 4. Section 5 is simulation experiments and physical experiments. Section 6 is the power consumption test. Conclusions are drawn in Section 7.

## 2. The Description of the Security Model

### 2.1. System Model

N sensor nodes are deployed in a target area to monitor a common physical state $\left(\theta_{0}/\theta_{1}\right)\;$ in a distributed manner. The measurement of each sensor $s_{i}$ is quantized into binaries (local decision $u_{i}$) based on the measurement and a decision threshold. If the measurement is less than the threshold, then $u_{i}=0$, otherwise $u_{i}=1$. Let $p(u_{i}=1|\theta_{1})$ and $p(u_{i}=1|\theta_{0})$ denote the local detection rate $p_{di}$ and false alarm rate $p_{fi}$ of the i-th sensor, respectively. Usually, $p_{di}$ and $p_{fi}$ can be known in advance both by the AFC and EFC.

**Figure 1:**
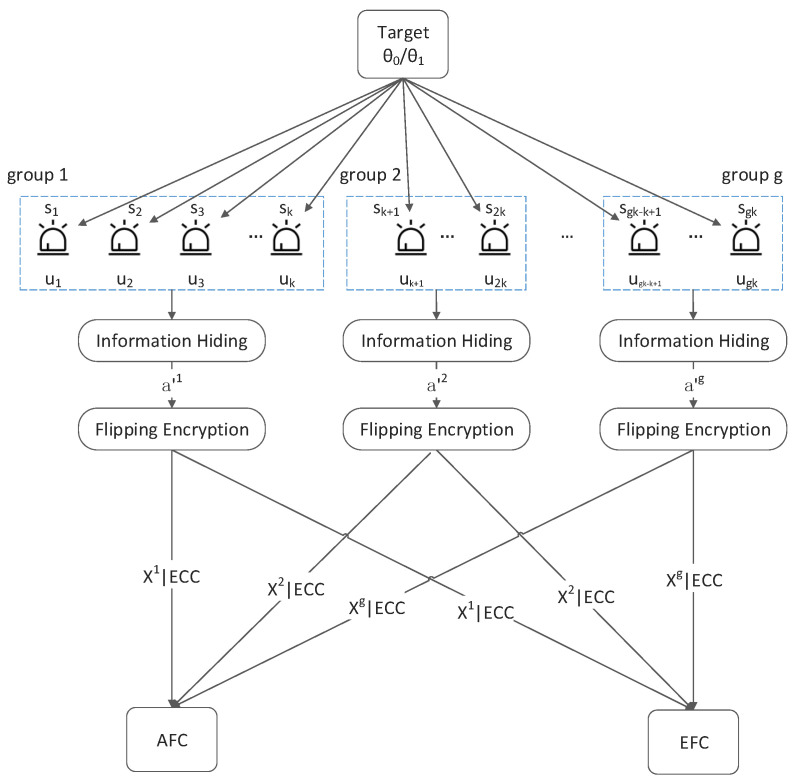
The framework of a wireless sensor network with an ally fusion center and an eavesdropping fusion center.

**Figure 2:**
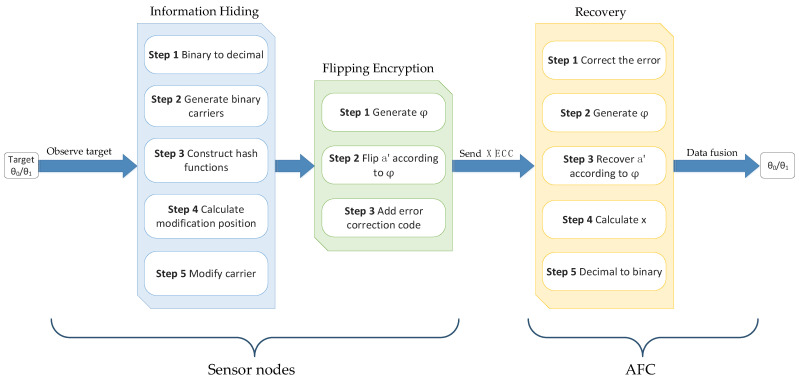
The main process of the proposed scheme.

The framework of the system discussed in this paper is shown in Figure 1. The N sensors are divided into g groups and each group comprises k sensors. The k decision bits from the corresponding *k* sensors are converted into one decimal number, x. Then, x is embedded in a customized binary string $a^{\prime }$ as hidden information through the F5 algorithm [16]. After that, $a^{\prime }$ is flipped according to the n-bit output sequence $\varphi =\varphi_{1}\;\varphi_{1}\ldots \;\varphi_{n}$ of a pseudo-random function rand(·) and threshold λ. ${a^{\prime }}_{i}$ is assigned to the flipping group if $\;\varphi_{i}$ is in the flipping domain, otherwise, it is assigned to the non-flipping group. Generally, the output sequence of rand(·) is decided by its initial seed. The flipped result $X^{j}$ of each group is sent to AFC along with its error correction code (ECC). In this paper, forward error correction (FEC) is employed to ensure that AFC can correct limited errors in the received data *X*. Finally, AFC recovers the flipped binary string, then extracts the embedded information x from the string, and obtains the real state of the target state by data fusion. The main process of the proposed method is shown in Figure 2.

### 2.2. Information Hiding and Recovery

Before data transmission, the measurements of each sensor are hidden into a binary string, which is called carrier in traditional digital steganography [16]. The steps for embedding the message are as follows:

Step 1 k sensors simultaneously observe the common target state and generate k binary observations $U=u_{1}u_{2}\ldots u_{k}$ and U is converted to a decimal number x.

Step 2 Randomly generate an n-bits binary carrier $a=a_{1}a_{2}\ldots a_{n}$.

Step 3 Construct a hash function f(·) using successive XOR ‘⊕’ operations on $a_{i}\;*\;i$, i=1,2…n, which is shown in Equation (1).

(1) $$f\left(a\right)=\;{⊕}_{i=1}^{n}a_{i}*i$$

Step 4 Calculate the position s of the binary bit to be flipped in a, 0 ≤ s ≤ n, as shown in Equation (2).

(2) s=x⊕f(a)

where *x* is the secret to be hidden in *a*.

Step 5 Modify a according to s, as shown in Equation (3).

(3) $$a^{\prime }=\{\begin{array}{ll} a, & if\;s=0\\ a_{1}a_{2}\ldots {\bar{a}}_{s}\ldots a_{n}, & if\;s\neq 0 \end{array}$$

where ${\bar{a}}_{s}=1⊕a_{s}$ denotes the inversion of the s-th bit in a.

Suppose the carrier embedding before and after is a and $a^{\prime },$ respectively, and the Hamming distance between a and $a^{\prime }$ is employed to represent the change caused by the modification of the carrier, which is shown in Equation (4).

(4) $$d\left(a,a^{\prime }\right)=\overset{n}{\underset{i=1}{\sum }}\left|a_{i}-{a^{\prime }}_{i}\right|\leq d_{max}$$

The triple $\left(d_{max},n,k\right)$ denoted that there are n modifiable positions in $a=a_{1}a_{2}\ldots a_{n}\;$ to embed k-bit secret messages. Usually, F5 [16] requires that at most one bit of *a* is modified to get *a*’, such that $d_{max}=1$. When we withdraw the encoded secret x from *a*’, the hash function $f\left(a^{\prime }\right)$ is calculated, and $x=f\left(a^{\prime }\right)$.

Equation (5) is a derivation to prove that the hash function satisfies $f\left(a^{\prime }\right)=x$.

(5) $$\begin{array}{l} f\left(a^{\prime }\right)=[{⊕}_{i=1,i\neq s}^{n}a_{i}*i]⊕[{\bar{a}}_{s}*s]\\ =⊕{[}_{i=1,i\neq s}^{n}a_{i}*i]⊕[\left(1⊕a_{s}\right)*\left(x⊕f\left(a\right)\right)]\\ =⊕{[}_{i=1,i\neq s}^{n}a_{i}*i]⊕[1*\left(x⊕f\left(a\right)\right)⊕a_{s}*\left(x⊕f\left(a\right)\right)]\\ =⊕{[}_{i=1,i\neq s}^{n}a_{i}*i]⊕[\left(x⊕f\left(a\right)\right)⊕a_{s}*\left(x⊕f\left(a\right)\right)]\\ =⊕{[}_{i=1,i\neq s}^{n}a_{i}*i]⊕\left(x⊕f\left(a\right)\right)⊕a_{s}*s\\ =⊕{[}_{i=1,i\neq s}^{n}a_{i}*i]⊕a_{s}*s⊕\left(x⊕f\left(a\right)\right)\\ ={⊕}_{i=1}^{n}a_{i}*i⊕x⊕f\left(a\right)\\ =f\left(a\right)⊕x⊕f\left(a\right)\\ =x \end{array}$$

Usually, n and k satisfy $n=2^{k}-1$. It is well known that a k-bit binary string has $2^{k}$ different permutations and can represent $2^{k}$ different numbers from 0 to $2^{k}-1$. Since the F5 algorithm requires the carrier to be modified by no more than 1 bit, the unmodified state of the carrier represents 0. Therefore, the carrier can represent the remaining $2^{k}-1$ numbers when its length is $2^{k}-1$.

Accordingly, the carrier modification density D(k) and the embedding rate R(k) can be calculated in Equations (6) and (7). R(k) represents how much information is hidden in a unit carrier. The lower the R(k), the lower the carrier utilization and the higher the security. With the same embedding capacity, a low embedding rate will use more carriers than a high embedding rate, and therefore the computational effort and power consumption of the sensor will increase.

(6) $$D\left(k\right)=\frac{1}{n+1}=\frac{1}{2^{k}}$$

(7) $$R\left(k\right)=\frac{k}{n}=\frac{1}{n}*\ln\left(n+1\right)=\frac{k}{2^{k}-1}$$

According to D(k) and R(k), the embedding efficiency W(k) can be defined in Equation (8). W(K) indicates how many bits can be embedded for each change of $a_{i}$, which is approximately equal to k. With the same embedding capacity, the higher the W(k), the less the modification to the carrier will be, but the computational load will increase instead. Moreover, whenever one bit of the carrier intercepted by the adversary is incorrect, a completely different k-bit data will be recovered. Therefore, the security decreases as W(k) increases.

(8) $$W\left(k\right)=\frac{R\left(k\right)}{D\left(k\right)}=\frac{2^{k}*k}{2^{k}-1}$$

Table 1 shows that the carrier data embedding rate R(k) decreases with the increase of embedding efficiency W(k). Therefore, to realize a balance between embedding rate and embedding efficiency, it is better to choose a shorter ciphertext length k.

### 2.3. Flipping Encryption

In the previous section, the outputs of k sensors were embedded in the n-bit string $a^{\prime }$. A pseudo-random function rand(.) is previously deployed at both the sensor nodes and AFC. The output of the function determines which bits of $a^{\prime }$ will be flipped. If the i-th output $\varphi_{i}$ of rand(.) falls within the flipping interval, then ${a^{\prime }}_{i}$ is flipped, otherwise, it is not flipped. As rand(.) is a pseudo-random function, if the AFC and the sensor have the same initial seed, the same random sequence can be generated synchronously at both sides. The seed can be determined by channel state information or received signal strength indicator (RSSI). Due to the channel independence, the EFC can only know the channel state from itself to the sensors, but nothing about the channel state from the AFC to the sensors because of the physical independence. Before the data transmission at each duplex time, the AFC first sends pilot signals $\left\{\tau_{1},\tau_{2},\tau_{3}\right\}$ to the sensor, where $\left\{\tau_{1}>\tau_{2}>\tau_{3}\right\}$. The sensor sends a local decision to the AFC after receiving the pilot channel. In this way, the AFC and the sensor initialize the same seed with RSSI. The sensor generates a new φ each time it receives the pilot signal, and the AFC generates the same φ after receiving the sensor signal. The pseudo-random function generates the results in the way shown in Equation (9). t refers to the t-th transmission of sensor data in a duplex time. The first φ is generated with RSSI as the seed, after which the previous φ is used as the seed to generate φ.

(9) $$\varphi_{i}^{\left(t\right)}=\{\begin{array}{c} rand\left(seed_{i}\right),\;\;\;\;\;\;\;\;\;if\;\;t=1\\ rand\left(\varphi_{i}^{\left(t-1\right)}\right),\;\;\;\;\;\;\;if\;\;t>1 \end{array}$$

The encrypted data of $a^{\prime }$ are denoted by X, where $X=X_{1}\ldots X_{n}$. If $\tau_{2}<\varphi_{i}<\tau_{1}$, $X_{i}$ will be put into the non-flipping group, that is, $X_{i}={a_{i}}^{\prime }$. If $\tau_{3}<\varphi_{i}<\tau_{2}$, $X_{i}$ will be put into the flipping group, that is, $X_{i}={a_{i}}^{\prime }$, that is $X_{i}=1⨁{a_{i}}^{\prime }$.

φ is manually set to obey a uniform distribution. For instance, we use the rand() function in MATLAB to generate random numbers that are uniformly distributed in the interval (0,1) in the simulation. Its probability density function is $f\left(x\right)=\frac{1}{\tau_{1}-\tau_{3}}$, therefore, its cumulative distribution function is shown in Equation (10).

(10) $$F\left(x\right)=\int_{\tau_{3}}^{\tau_{2}}f\left(x\right)dx=\int_{\tau_{3}}^{\tau_{2}}\frac{1}{\tau_{1}-\tau_{3}}dx$$

We use λ to denote the probability that ${a_{i}}^{\prime }$ is flipped. If the flipping probability is λ, then the probability of not flipping is 1−λ. The definition of λ is shown in Equation (11).

(11) $$\lambda =\frac{\tau_{2}-\tau_{3}}{\tau_{1}-\tau_{3}}$$

Then, we encode X with the BCH error correction code (ECC) and send X|ECC to the AFC. In the proposed scheme, the AFC might not recover $a^{\prime }$ correctly if there is channel noise. As long as one bit is wrong, completely incorrect sensor data will be recovered. Error correction codes can increase the robustness and improve the probability of correct data recovery. BCH code is a classical and effective forward error correction code that corrects limited errors immediately as the data are received. For example, the (7,4,3) BCH code used in the experiment, which encodes 4 bits of data into 7 bits, can correct 1 bit of error.

Due to the broadcast nature of WSN, the EFC can eavesdrop on messages that AFC can receive. However, it cannot distinguish between flipped data and unflipped data, let alone extract the original sensor outputs embedded in it. Therefore, the EFC is completely unable to use the information it eavesdrops on.

### 2.4. The Fusion Result of the AFC

In this section, the fusion result of the AFC based on the log-likelihood ratio (LLR) is analyzed. Assume that the output vectors of N sensors received by AFC are $z=\left[z_{1}^{A}\ldots z_{N}^{A}\right]$, and according to [17] the LLR-based fusion rule at the AFC is given by Equation (12):

(12) $$\Lambda =log\frac{P(z^{A}|\theta_{1})}{P(z^{A}|\theta_{0})}=\overset{N}{\underset{i=1}{\sum }}log\frac{f(z_{i}^{A}|\theta_{1})}{f(z_{i}^{A}|\theta_{0})}$$

The main purpose of multi-sensor fusion decision is to obtain detection performance that is not achievable by any single sensor by fusing the detection results of multiple sensors. The fusion results are compared with the decision threshold, which affects the accuracy of the fused sensor data. How to find the optimal threshold is a problem that many researchers are exploring, but it is not the focus of this paper. Therefore, in the experiments of this paper, the decision threshold is set to 0. If Λ is greater than the decision threshold, the fusion decision result is $\theta_{1}$, otherwise, it is $\theta_{0}$. f(·|·) is the conditional probability density function of the sensor. The sensors’ measurements are assumed to be independent and identically distributed (i.i.d.) under $\theta_{0}/\theta_{1}$.

In Section 2.2 and Section 2.3, N sensors have been divided into g groups. The k sensor outputs of each group have been evenly embedded in the binary string $a^{\prime }=\left[a_{1}\ldots a_{n}\right]$, which are then flipped and encrypted into the binary string $X=X_{1}\ldots X_{n}$. After receiving the data, the AFC first corrects errors in the transmission by FEC and then recovers $a^{\prime }$ via Equation (13).

(13) $${a_{i}}^{A^{\prime }}=\{\begin{array}{l} \;\;X_{i}^{A},\;\;\;\;\;\;\;\;\;if\;\;\;\tau_{2}<\varphi_{i}=rand\left(\varphi_{i}^{t-1}\right)<\tau_{1}\\ 1⨁X_{i}^{A},\;\;\;\;\;\;if\;\;\;\tau_{3}<\varphi_{i}=rand\left(\varphi_{i}^{t-1}\right)<\tau_{2}\;\; \end{array}$$

AFC can easily recover ${a_{i}}^{A^{\prime }}$ and calculate $f\left(a^{A^{\prime }}\right)=x^{A}$. $x^{A}$ can be converted to the binary string $z_{1}^{A}\ldots z_{k}^{A}$. In the same way, we can recover the sensor output of each group and get the output vector of N sensors $z^{A}=z_{1}^{A}\ldots z_{N}^{A}$. Let $U_{1}=\{i|z_{i}^{A}=1\}$ and $U_{2}=\left\{i|z_{i}^{A}=0\right\},$ then the fusion rule can be rewritten as Equation (14):

(14) $$\Lambda =\underset{i\in U_{1}}{\sum }log\frac{f(z^{A}|\theta_{1})}{f(z^{A}|\theta_{0})}+\underset{i\in U_{2}}{\sum }log\frac{f(z^{A}|\theta_{1})}{f(z^{A}|\theta_{0})}$$

where

(15) $$\underset{i\in U_{1}}{\sum }log\frac{f(z^{A}|\theta_{1})}{f(z^{A}|\theta_{0})}=\underset{i\in U_{1}}{\sum }log\frac{f(u_{i}{}^{A}=1|\theta_{1})}{f(u_{i}{}^{A}=1|\theta_{0})}=\underset{i\in U_{1}}{\sum }log\frac{pd_{i}}{pf_{i}}$$

(16) $$\underset{i\in U_{2}}{\sum }log\frac{f(z^{A}|\theta_{1})}{f(z^{A}|\theta_{0})}=\underset{i\in U_{1}}{\sum }log\frac{f(u_{i}{}^{A}=0|\theta_{1})}{f(u_{i}{}^{A}=0|\theta_{0})}=\underset{i\in U_{1}}{\sum }log\frac{1-pd_{i}}{1-pf_{i}}$$

Therefore, the final fusion rule is shown in Equation (17).

(17) $$\Lambda =\underset{i\in U_{1}}{\sum }log\frac{pd_{i}}{pf_{i}}+\underset{i\in U_{1}}{\sum }log\frac{1-pd_{i}}{1-pf_{i}}$$

If Λ is greater than the decision threshold, the fusion decision result is $\theta_{1}$, otherwise, it is $\theta_{0}$. Note that this approximation can be regarded as a modified version of the Chair–Varshney fusion rule, which utilizes only the local detection rate and false alarm rate.

## 3. Security Analysis

In this section, we analyze the security of the scheme by discussing whether the EFC can obtain useful information from the eavesdropped data. The data eavesdropped by EFC from the sensor nodes are $X^{E}=X_{1}{}^{E}\ldots X_{n}{}^{E}$. Since the EFC does not know the channel status of the AFC and the common seed of the sensor and the AFC, it can only use the RSSI of the eavesdropped data as the seed. It recovers ${a_{i}}^{E^{\prime }}$ as follows:

(18) $${a_{i}}^{E^{\prime }}=\{\begin{array}{c} X_{i}^{E},\;\;\;\;\;\;\;\;\;\;\;\;\;if\;\;\;\tau_{2}<\varphi_{i}{}^{E}=rand\left(\varphi_{i}^{t-1}{}^{E}\right)<\tau_{1}\\ 1⨁X_{i}^{E},\;\;\;\;\;\;\;\;if\;\;\;\tau_{3}<\varphi_{i}{}^{E}=rand\left(\varphi_{i}^{t-1}E\right)<\tau_{2}\;\; \end{array}$$

Next, we intend to prove $f\left(a^{E^{\prime }}\right)=x^{E}\neq x$. It may be assumed that there are m bits in $a^{E^{\prime }}$ that cannot be recovered correctly and $S=\{i|a^{E^{\prime }}=1⊕{a_{i}}^{A^{\prime }}\}$. If $f\left(a^{E^{\prime }}\right)=f\left(a^{A^{\prime }}\right)$, then $f\left(a^{E^{\prime }}\right)⊕f\left(a^{A^{\prime }}\right)=0$. Thus

(19) $$\begin{array}{l} f\left(a^{E^{\prime }}\right)⊕f\left(a^{A^{\prime }}\right)=⊕{[}_{i=1,i\notin S}^{n}{a_{i}}^{E}*i]⊕{[}_{i\in S}a_{i}{}^{E}*i]⊕{[}_{i=1}^{n}a_{i}*i]\\ =⊕{[}_{i=1,i\notin S}^{n}a_{i}{}^{E}*i]⊕{[}_{i\in S}{ai}^{E}*i]⊕{[}_{i=1,i\notin S}^{n}a_{i}{}^{A}*i]⊕{[}_{i\in S}a_{i}{}^{A}*i]\\ =⊕{[}_{i\in S}a_{i}{}^{E}*i]⊕{[}_{i\in S}a_{i}{}^{A}*i]\\ =⊕{[}_{i\in S}(1⊕a_{i}{}^{A})*i]⊕{[}_{i\in S}a_{i}{}^{A}*i]\\ =⊕{[}_{i\in S}1*i{]⊕[}_{i\in S}a_{i}{}^{A}*i]⊕{[}_{i\in S}a_{i}{}^{A}*i]\\ =⊕{[}_{i\in S}1*i]\\ =⊕{[}_{i\in S}i] \end{array}$$

(20) $$\begin{array}{l} f\left(a^{E^{\prime }}\right)=⊕{[}_{i\in S}i]⊕f\left(a^{A^{\prime }}\right)\\ =⊕{[}_{i\in S}i]⊕x=x^{E} \end{array}$$

It can be inferred that $f\left(a^{E^{\prime }}\right)⊕f\left(a^{A^{\prime }}\right)\neq 0$, unless $⊕{[}_{i\in S}i]=0$.

$x^{E}$ can be converted to a binary string $z_{1}^{E}\ldots z_{k}^{E}$. Next, we can prove that the probabilities of $z_{i}^{E}=z_{i}^{A}$ and $z_{i}^{E}=-z_{i}^{A}$ are equal.

(21) $$P\left(z_{i}^{E}=z_{i}^{A}\right)=\frac{C_{m}^{0}+C_{m}^{2}+C_{m}^{4}\ldots }{2^{m}}=\frac{\sum_{j=2n}C_{m}^{j}}{2^{m}}$$

(22) $$P\left(z_{i}^{E}=-z_{i}^{A}\right)=\frac{C_{m}^{1}+C_{m}^{3}+C_{m}^{5}\ldots }{2^{m}}=\frac{\sum_{j=2n+1}C_{m}^{j}}{2^{m}}$$

From a statistical point of view, m=λ·n. Obviously, when *m* > 1, i.e., λ>1/n, there is $P\left(z_{i}^{E}=z_{i}^{A}\right)=P\left(z_{i}^{E}=-z_{i}^{A}\right)=0.5$.

Similarly, we can recover the sensor output of each group to obtain the output vector of N sensors $x^{E}=z_{1}^{E}\ldots z_{N}^{E}$. Then, the fusion rule of EFC can be expressed as:

(23) $$L=log\frac{f(z^{E}|\theta_{1})}{f(z^{E}|\theta_{0})}$$

If L is greater than the decision threshold, the fusion decision result is $\theta_{1}$, otherwise, it is $\theta_{0}$. It can be seen from Equation (23) that data confidentiality can be achieved by deriving $f\left(z^{E}|\theta_{1}\right)$ equal to $f(z^{E}|\theta_{0})$, which makes the LLR value at the EFC always equal to zero. The EFC will ignore the data since it cannot make a final decision for the binary hypothesis testing problem when L=0.

(24) $$\begin{array}{l} f\left(z^{E}|\theta_{1}\right)=P\left(z^{A}=1|\theta_{1}\right)\cdot P\left(z_{i}^{E}=z_{i}^{A}\right)+P\left(z^{A}=0|\theta_{1}\right)\cdot P\left(z_{i}^{E}=-z_{i}^{A}\right)\\ \;\;\;\;\;=pd\cdot P\left(z_{i}^{E}=z_{i}^{A}\right)+\left(1-pd\right)\cdot P\left(z_{i}^{E}=-z_{i}^{A}\right)=0.5 \end{array}$$

Similarly,

(25) $$\begin{array}{l} f\left(z^{E}|\theta_{0}\right)=P\left(z^{A}=0|\theta_{0}\right)\cdot P\left(z_{i}^{E}=z_{i}^{A}\right)+P\left(z^{A}=1|\theta_{0}\right)\cdot P\left(z_{i}^{E}=-z_{i}^{A}\right)\\ \;\;\;\;\;=\left(1-pf\right)\cdot P\left(z_{i}^{E}=z_{i}^{A}\right)+pf\cdot P\left(z_{i}^{E}=-z_{i}^{A}\right)=0.5 \end{array}$$

Obviously,

(26) $$L=log\frac{f(z^{E}|\theta_{1})}{f(z^{E}|\theta_{0})}=0$$

As can be deduced from Equation (26), the LLR at EFC is always equal to 0, which makes it impossible to decide the target state. Therefore, it is almost impossible for EFC to make correct decisions based on the captured sensor data.

## 4. Time Complexity Evaluation

In this section, the time complexity of this scheme in a duplex time is analyzed. To compare with traditional encryption algorithms, we define the stage from the sensors observing target state to sending data as the encryption stage, and the stage from the AFC receiving data to taking out the sensor outputs as the decryption stage. Then, we use Big O notation to describe the time complexity of the encryption stage and the decryption stage. The proposed scheme in this paper is referred to as the information hiding and data flipping (IHDF) scheme.

In step 1 of Section 2.2, the time complexity of N sensors to observe the target state is O(N). Since N=gk, O(N)→O(gk). In step 2, the time complexity of generating a random binary string a of length n is O(gn). In step 3, the time complexity of calculating the hash function f(a) is also O(gn). In step 4, the time complexity of calculating the position s of the bits in a to be changed is O(g). In step 5, the time complexity of the operation of modifying a according to s at most one bit is O(g). In Section 2.3, the time complexity of the sensor generating φ that determines whether a’ is flipped is O(gn). The time complexity of calculating the error correction code of the encrypted X is O(gn).Therefore, the time complexity of the encryption stage of IHDF is O(gk+4gn+2g)→O( N/k·(k+4n+2))→O(N/R(x)).

Similarly, the time complexity of AFC for error correction of the received messages is O(gn). The time complexity of generating the flipping sequence φ is O(gn). The time complexity of calculating $a^{\prime }$ is O(gn). The time complexity of calculating $x=f\left(a^{\prime }\right)$ is O(gn). The time complexity of restoring x to binary output is O(gk). Therefore, the time complexity of the decryption stage of IHDF is O(5gn+gk)→O(N/k·(5n+k))→O(N/R(x)).

The computation load of the sensor nodes is O(N/R(x)) and the computation load of the AFC is also O(N/R(x)). The time complexity decreases as R(x) increases.

RC6 [18], Simon [19], and Speck [19] are typical lightweight encryption algorithms, while Rijndael [20] and Twofish [21] are traditional symmetric encryption algorithms. Table 2 shows the computational complexity of each algorithm. Compared with Rijndael and Twofish, the proposed scheme has no complicated S-box confusion, and the time and space costs are significantly reduced. For symmetric encryption algorithms, if the information to be encrypted is too short, such as a few bits, it must be filled to at least the shortest encryption length. At the same time, multiple iterative calculations are required, which will increase the computational effort.

## 5. Experiments

### 5.1. Simulation Result

In this section, a group of simulations is performed to demonstrate that the AFC can correctly fuse the sensor outputs when there are only two candidate states, while EFC cannot eavesdrop on the real state.

The comparison object is the method in [10] in which the sensor randomly flips its output according to the channel gain, and the AFC calculates the true state through the channel statistics. In addition, the performance of the Optimum-LLR proposed in [22], where the Optimum-LLR-based fusion rule is derived within the absence of the EFC, is considered as the lower bounds of the error probability.

In the simulation experiment, for comparison, we used a similar condition as in [1], deploying the sensors in a star-like topology and assuming that the main channel gain follows the Rayleigh distribution. The total error probability by $P_{\varepsilon }=\delta \left(1-P_{d}\right)+\left(1-\delta \right)P_{f}$ is taken as the criterion of fusion performance, where $P_{d}$ and $P_{f}$ are the detection and false alarm probabilities at the fusion center. Furthermore, the sensors have the same local detection performances, $P_{d}=0.8$ and $P_{f}=0.2$. In addition, δ is the weighting factor and we set δ=0.5. In our scheme, there are five sensors in a group, i.e., k=5 and n=31, and we set the flip probability λ=0.5. Otherwise, we use (7,4,3) Bose–Chaudhuri–Hocquenghem (BCH) codes as the error correction code. All parameters are shown in Table 3.

**Figure 3:**
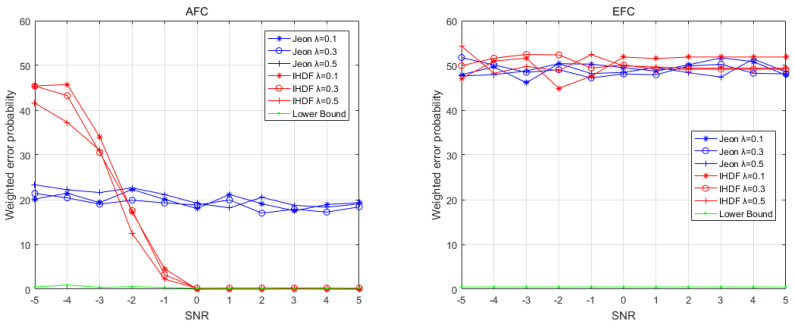
The weighted error probabilities at the AFC and EFC as a function of SNR in ideal channel conditions.

**Figure 4:**
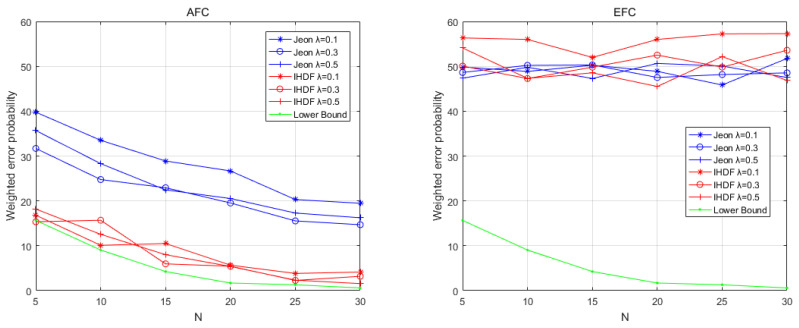
The weighted error probabilities at the AFC and EFC with an increasing number of sensors in ideal channel conditions.

The first round of comparisons is performed under the ideal conditions that the channel gains strictly follow the Rayleigh distribution. The results are shown in Figure 3 and Figure 4, which depict the weighted error probability (WEP) of the AFC and EFC with a different signal-to-noise ratio (SNR) and numbers of sensors. Figure 3 shows the WEP of AFC and EFC as the SNR increases when the number of sensors is 20. It can be seen that under ideal channel conditions, the AFC using IHDF can obtain a lower error probability than Jeon’s method when the SNR is greater than −2, even approaching the lower bound after the SNR is greater than 0. Figure 4 shows the WEP of AFC and EFC as the number of sensors increases when the SNR is −1. It can be seen that in an ideal channel, the AFC error probability of IHDF is very close to the lower bound, which is significantly lower than that of Jeon’s method. As can be seen from Figure 3 and Figure 4, the error probability of EFC is always around 50%. Obviously, under ideal conditions, both IHDF and Jeon can achieve complete confidentiality of information.

To further test the proposed scheme, we designed another group of comparisons that simulates a more realistic network environment in which the channel gain varies continuously and we have no prior knowledge of the probability distribution of the gain.

**Figure 5:**
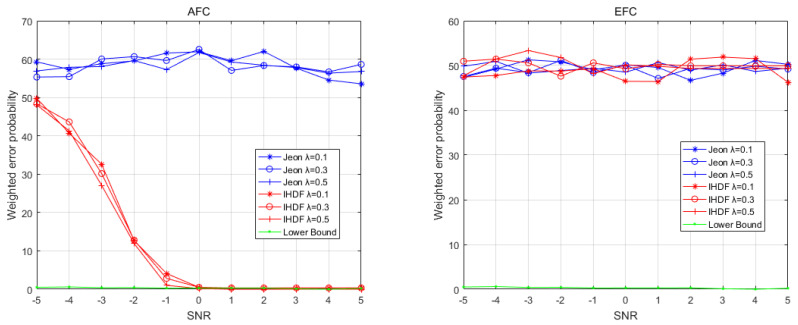
The weighted error probabilities at the AFC and EFC as a function of SNR in time-varying channel conditions.

**Figure 6:**
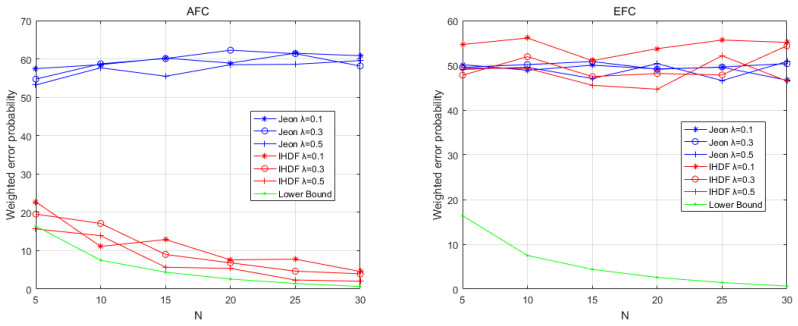
The weighted error probabilities at the AFC and EFC with an increasing number of sensors in time-varying channel conditions.5.2. Physical Experiment Results.

Figure 5 shows the WEP of AFC and EFC as SNR increases in a time-varying environment when the number of sensors is 20. It can be seen that the AFC using IHDF can obtain an error probability close to the lower bound when the SNR is greater than −2 under time-varying environmental conditions. The error probability of Jeon’s method will exceed 50%. Figure 6 shows the WEP of AFC and EFC as the number of sensors increases when the SNR is −1 under time-varying environmental conditions. IHDF can reduce the error probability as the number of sensors increases, but Jeon’s error probability remains at around 50%. However, EFC still cannot distinguish between flipped and unflipped data with an error probability of about 50%. Obviously, in a time-varying environment, although Jeon’s method can guarantee the confidentiality of information, it has been completely unable to restore the original data. The proposed scheme is more resistant to changes in channel conditions than Jeon’s method and can guarantee complete confidentiality of information while maintaining a lower error probability.

### 5.2. Physical Experiment Results

**Figure 7:**
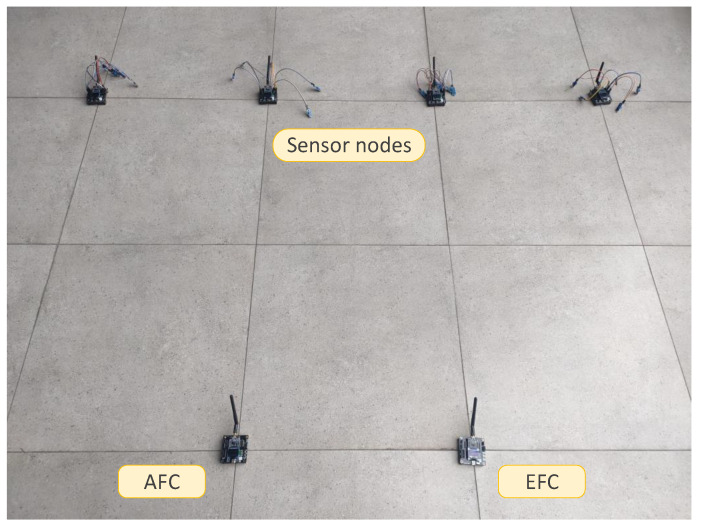
Sensor devices in the experiment.

**Figure 8:**
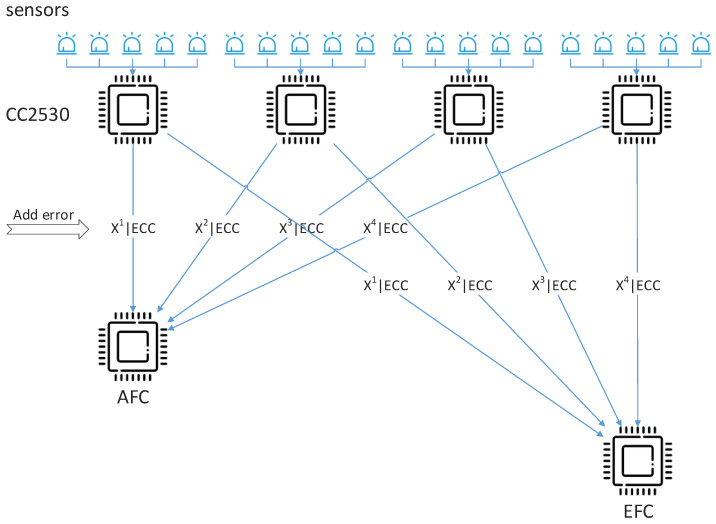
Experimental topology.

In this section, we experiment with the method in a real physical environment. We prepared six CC2530 development boards, two of which are used as AFC and EFC, respectively, and the other four are equipped with five photosensitive sensors each. The current presence or absence of light is regarded as the physical target for common observation. Firstly, the AFC or EFC is connected to our computer through a USB cable, and then the collected data from the sensors by the AFC and EFC along with their fusion results are read out by serial debugging software in real time, respectively. The sensors’ local detection rate and false alarm rate are 0.95 and 0.1, respectively, which are known both by the AFC and EFC. The rest of the parameters remain the same as in Section 5.1. The parameters of the sensor devices are shown in Table 4. The experimental devices and the topology of the physical sensor network in our experiments are shown in Figure 7 and Figure 8, respectively.

The comparison objects are three lightweight symmetric encryption algorithms: RC6, Simon, and Speck. To meet the low power consumption requirements of WSN, the calculation process should be as simple as possible. Therefore, we selected the lowest encryption bits and the shortest key length of these algorithms to encrypt the sensor output directly and decrypt it in the fusion center. After artificially adding a certain percentage of error bits to the data sent from the sensor to the fusion center, we measured the error probabilities of AFC and EFC as the number of sensors increased. Each compared algorithm is running in the sensor network for at least one hour to generate more than 5800 pieces of sensor data.

**Figure 9:**
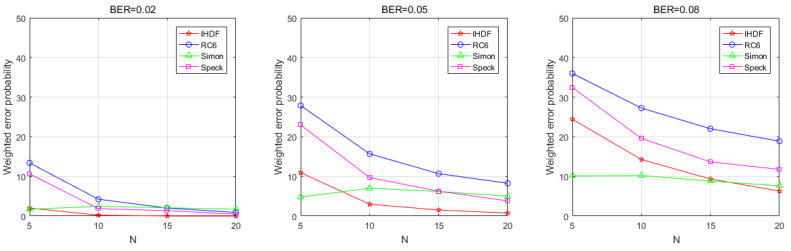
The weighted error probabilities at the AFC with an increasing number of sensors in the physical environment.

**Figure 10:**
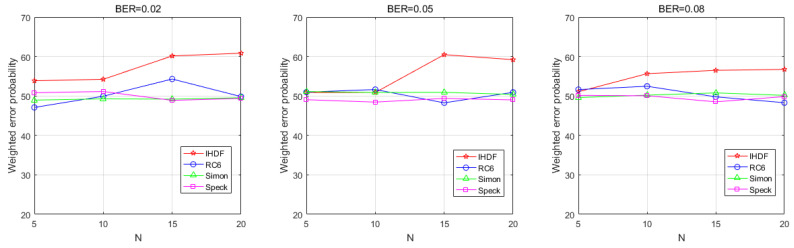
The weighted error probabilities at the EFC with an increasing number of sensors in the physical environment.

Figure 9 shows that the WEP of the AFC decreases as the number of sensors increases in the physical WSN environment. The WEP of the IHDF decreases as the number of sensors increases. At a BER of 0.2, the error probability of the IHDF remains in a very low range. At a BER of 0.5, the error probability can be reduced to less than 5% by increasing the number of sensors, which makes the error probability of IHDF significantly lower than that of other algorithms. This is because the error correction mechanism of IHDF enables AFC to actively correct the error of the received codeword when receiving sensor data. Generally, sensors in WSN are battery-powered, but AFC is usually powered by an external power source, so the operation of correcting the incorrect code would not add too much burden to the AFC. Even at a BER of 0.08, the error probability can be reduced to less than 10% by increasing the number of sensors. Compared with other algorithms, the proposed scheme outperforms RC6 and Speck and is only slightly inferior to Simon in the case of high BER. Figure 10 shows that in a physical network, the EFC is unable to distinguish between flipped and unflipped data when eavesdropping on the communication data between the sensor and AFC, with an error probability of about 50%.

As can be seen from Figure 9 and Figure 10, IHDF utilized steganography and random flipping to ensure the data confidentiality, which has higher anti-interference ability and efficiency, while ensuring the confidentiality as the lightweight symmetric encryption algorithm.

## 6. Power Consumption Evaluation

In this section, we measured the power consumption of the algorithm in Section 5.2 after running on the CC2530 development board for a while.

We put the fully charged 14,500 lithium battery into the CC2530 development board and send the encrypted sensor output at a rate of 5800 times per hour. After the development board continues to run the algorithm for several hours, the remaining battery power is measured. We continue to discharge the battery with a current of 1 A. The discharge ends when the battery voltage drops to the cutoff voltage. The total amount of discharge during this time is the remaining charge of the battery. Similarly, the fully charged battery power can be measured. The hourly power consumption is calculated by dividing the difference between the fully charged battery power and the remaining power by the operating time.

To avoid errors caused by different development boards and batteries, we ran each algorithm once on each of the four development boards, which corresponded to the batteries one by one. To measure the pure power consumption of the algorithm, all sensors were removed from the development board before starting the measurement. The average of the four measurements was then taken as the final hourly power consumption. The measurement order and measurement values for each algorithm are shown in Table 5. It can be seen that the proposed scheme consumes significantly less power compared with the traditional lightweight symmetric encryption algorithm. Combined with Table 2, the proposed scheme has no iterative computation, only 5 bits of data are encrypted, and the carrier bit string used to hide the information is only 31 bits. The amount of running code is much smaller than that of the traditional symbolic measure encryption algorithm. Therefore, the proposed scheme is more suitable than the traditional method for wireless sensor network applications that require low energy consumption.

## 7. Conclusions

In this paper, a lightweight scheme based on information hiding is proposed for data confidentiality in distributed WSNs. Due to the openness of WSNs, sensor data are transmitted through insecure channels. The EFC can eavesdrop on all transmitted data from sensors and calculate the state of the observed target through data fusion. To prevent the eavesdropping of EFC, we designed a transmission scheme based on information hiding and data flipping. The main idea is to hide the local measurements into a customized binary string based on the principle of encoding hiding. Then, the sensors and the AFC generate a sequence of random numbers synchronously based on the output of a pre-deployed pseudo-random function, which determines the data flipping. Finally, the flipped binary strings are sent to the AFC along with error correction codes. The AFC can recover the flipped string and then extract the sensor’s observations from the string.

Through simulations and experiments in real physical equipment, it is demonstrated that the proposed scheme enables the AFC to correctly recover the sensor output, while the EFC is completely unable to distinguish between flipped and unflipped data. The proposed scheme has good immunity to interference and can still restore the original data more accurately in the changing physical environment. Moreover, the proposed method is more efficient through real power consumption tests and consumes less power than the traditional symmetric encryption.

We believe that the proposed scheme can be deployed in many WSN applications with limited resources and unpredictable operating environments, including natural disaster monitoring and remote control of UAVs, due to its anti-interference, low complexity, and low power characteristics.

## Figures and Tables

**Table 1:** The relationship between (k,n) and modification density, embedding rate, and embedding efficiency.

k	n	D(k)	R(k)	W(k)
1	1	50%	100%	2
2	3	25%	66.67%	2.67
3	7	12.50%	42.86%	3.43
4	15	6.25%	26.67%	4.27
5	31	3.12%	16.13%	5.16
6	63	1.56%	9.52%	6.09
7	127	0.78%	5.51%	7.06
8	255	0.39%	3.14%	8.03
9	511	0.20%	1.76%	9.02

**Table 2:** The computational complexity of different algorithms.

Algorithm	S-Box Confusion	Shortest Encryption Length (bit)	Shortest Key Length (bit)	Shortest Iteration Frequency
IHDF	no	1	0	1
RC6 [18]	no	128	128	20
Simon [19]	no	32	64	32
Speck [19]	no	32	64	22
Rijndael [20]	yes	128	128	10
Twofish [21]	yes	128	128	16

**Table 3:** Parameter setting.

Parameters	Value
Detection rate	0.8
False alarm rate	0.2
δ	0.5
k	5
n	31
λ	0.5
BCH codes	(7,4,3)

**Table 4:** The parameters of the sensor devices.

Parameters	Value
Chip	CC2530
Interface	USB/UART/SPI
Transmission speed	9600 Kbps
Operating frequency band	2.4 GHz
Operating Voltage	3.7 V
Transmission Protocol	Zigbee
Detection rate	0.95
False alarm rate	0.1
δ	0.5
k	5
n	31
λ	0.5
BCH codes	(7,4,3)

**Table 5:** Hourly power consumption of different algorithms.

Algorithm	Hourly Power Consumption (mWh/h)
IHDF	55
RC6	78
Simon32/64	61
Speck	68
Not encrypted	42
